# High tissue MMP14 expression predicts worse survival in gastric cancer, particularly with a low PROX1

**DOI:** 10.1002/cam4.2576

**Published:** 2019-09-27

**Authors:** Aaro Kasurinen, Silvia Gramolelli, Jaana Hagström, Alli Laitinen, Arto Kokkola, Yuichiro Miki, Kaisa Lehti, Masakazu Yashiro, Päivi M. Ojala, Camilla Böckelman, Caj Haglund

**Affiliations:** ^1^ Translational Cancer Medicine Research Program Faculty of Medicine University of Helsinki Helsinki Finland; ^2^ Department of Pathology and Oral Pathology University of Helsinki and Helsinki University Hospital Helsinki Finland; ^3^ Department of Surgery University of Helsinki and Helsinki University Hospital Helsinki Finland; ^4^ Department of Microbiology, Tumor and Cell Biology Karolinska Institute Stockholm Sweden; ^5^ Individualized Drug Therapy Research Program Faculty of Medicine University of Helsinki Helsinki Finland; ^6^ Department of Surgical Oncology Osaka City University Graduate School of Medicine Osaka Japan; ^7^ Section of Virology Division of Infectious Diseases Department of Medicine Imperial College London London UK

**Keywords:** gastric cancer, matrix metalloproteinase 14, prognosis, prospero homeobox protein 1, survival

## Abstract

Matrix metalloproteinase 14 (MMP14), a membrane‐associated matrix metalloproteinase, has been shown to influence the invasion and metastasis of several solid tumors. Prospero homeobox protein 1 (PROX1), involved in the development and cell fate determination, is also expressed in malignant diseases functioning either as a tumor‐suppressing or oncogenic factor. In certain cancers PROX1 appears to transcriptionally suppress MMP14 expression. This study, therefore, aimed to explore the association between MMP14 and PROX1 and understand their potential as prognostic biomarkers in gastric cancer. The cohort consisted of 313 individuals operated for gastric adenocarcinoma between 2000 and 2009 in the Department of Surgery, Helsinki University Hospital. MMP14 and PROX1 expressions were studied using immunohistochemistry in the patient sample and using immunoblotting and immunofluorescence in gastric cancer cell lines. We generated survival curves using the Kaplan‐Meier method, determining significance via the log‐rank test. A high MMP14 expression associated with being ≥67 years (*P* = .041), while a positive nuclear PROX1 expression associated with tumors of a diffuse histological type (*P* = .041) and a high cytoplasmic PROX1 expression (*P* < .001). Five‐year disease‐specific survival among patients with a high MMP14 expression was 35.9% (95% confidence interval [CI] 24.9‐46.9), compared to 45.3% (95% CI 38.0‐52.6) for patients with a low MMP14 (*P* = .030). Survival was worse specifically among those with a high MMP14 and absent nuclear PROX1 expression (hazard ratio [HR] 1.65; 95% CI 1.09‐2.51; *P* = .019). Thus, this study confirms that a high MMP14 expression predicts a worse survival in gastric cancer, revealing for the first time that survival is particularly worse when PROX1 is low.

## Graphical abstract

The aim of this study was to explore the association between matrix metalloproteinase 14 (MMP14) and prospero homeobox protein 1 (PROX1) and understand their potential as prognostic biomarkers in gastric cancer. MMP14 and PROX1 expressions were studied using immunohistochemistry in the patient samples and using immunoblotting and immunofluorescence in gastric cancer cell lines. This study confirms that a high MMP14 expression predicts a worse survival in gastric cancer, revealing for the first time that survival is particularly worse when PROX1 is low.

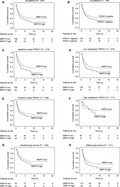

## 1 INTRODUCTION

Gastric cancer is one of the most common malignancies worldwide, featuring a poor prognosis largely due to its late diagnosis. Molecular classifications of the disease, in addition to current histological classifications, are essential for predicting the disease's behavior, the further development of therapy, and individualizing treatment. The Cancer Genome Atlas project proposed a novel molecular subtype classification, although it is not yet applicable for routine use.^1^, ^2^

Matrix metalloproteinases (MMPs), a group of zinc‐containing proteases expressed in a plethora of tissues, represent important contributors to metastasis due to their capabilities of degrading the extracellular matrix (ECM). Matrix metalloproteinases can also promote carcinogenesis by activating cell migration and oncogenic signaling pathways.^3^, ^4^ Previous studies demonstrated that a high expression of MMPs associated with a poor patient prognosis, tumor invasion, and metastasis in several cancers.^5^, ^6^, ^7^, ^8^, ^9^, ^10^

Matrix metalloproteinase 14 (MMP14), a membrane‐anchored MMP, features strong ECM‐degrading capabilities. Furthermore, MMP14 expression appears to be higher in gastric cancer tissues compared to noncancerous mucosa, indicating a worse prognosis in cancer patients with a high MMP14 expression compared to patients with a low MMP14 expression.^5^, ^9^, ^10^, ^11^

Prospero homeobox protein 1 (PROX1), a transcription factor related to organ development and cell fate determination,^12^, ^13^, ^14^, ^15^ is also expressed in various cancers acting either as a tumor suppressor or as an oncogene depending on the tumor type.^16^ In healthy gastric mucosa, miR‐489 inhibits PROX1 levels, while the loss of function mutations in gastric carcinogenesis directly results in increased PROX1 levels.^17^ Overexpression experiments involving miR‐489 resulted in the deceleration of growth and reduced PROX1 levels, suggesting that PROX1 may contribute to gastric carcinogenesis. However, a high immunohistochemical PROX1 expression in gastric cancer patients' tissue samples has been paradoxically linked both to better and worse prognoses.^18^, ^19^, ^20^

A functional link between MMP14 and PROX1 was recently established.^21^ The expression of PROX1 and MMP14 was, however, inversely related in cell lines and murine models, and additional experiments demonstrated that PROX1 directly inhibited the transcription of MMP14 by binding to its promoter region. Cellular mechanisms were investigated in several cell types, although not in gastric cancer cells.

Therefore, this study aimed to investigate the relationship between MMP14 and PROX1 in gastric cancer and to explore their values as prognostic biomarkers. We studied MMP14 and PROX1 expression levels in tissue samples compared with clinical data, and determined the MMP14 and PROX1 expression levels in gastric cancer cells.

## 2 MATERIALS AND METHODS

### 2.1 Patients

The cohort consisted of 313 individual patients operated for histologically verified gastric adenocarcinoma in the Department of Surgery, Helsinki University Hospital, between 2000 and 2009. We excluded patients with a history of other malignant disease or synchronous cancer. The median age at the time of surgery was 67.4 years (interquartile range [IQR] 57.1‐76.5) and 161 patients (51.4%) were female. The seventh version of the tumor‐node‐metastasis classification (TNM)^22^ was used for cancer staging: 62 (19.8%) were stage I, 72 (23.0%) were stage II, 115 (36.7%) were stage III, and 63 (20.1%) were stage IV. A total gastrectomy was required for 153 patients (48.9%) and partial for 160 (51.1%); lymphadenectomy was performed according to D1 in 107 (34.2%) and D2 in 203 patients (64.9%). In 198 patients (63.3%), the disease was lymph node‐positive and 63 cases (20.1%) also presented with distant metastases. According to the Laurén classification,^23^ 124 tumors (39.6%) were of the intestinal histological type. Curative surgery was possible for 228 patients (72.8%). In addition, 15 patients (4.8%) received neoadjuvant treatment and 125 patients (39.9%) received postoperative adjuvant treatment. The median follow‐up time was 2.3 years, with 66 patients (21.1%) still living at the end of follow‐up. Five‐year disease‐specific survival for the entire patient cohort was 43.3% (95% confidence interval [CI] 37.4‐49.2).

Living data and causes of death until September 2017 were acquired from patient records, the Population Register Center of Finland, and Statistics Finland.

The Surgical Ethics Committee of the Helsinki University Hospital (Dnro HUS 226/E6/06, extension TMK02 §66 17.04.2013) approved the study and study protocol. Authorization to use tissue samples retrospectively without individual consent was granted by the National Supervisory Authority of Welfare and Health (Valvira Dnro 10041/06.01.03.01/2012).

### 2.2 Sample preparation and immunohistochemistry

Original tumor samples were fixed in formalin, embedded in paraffin, and stored at the Department of Pathology, University of Helsinki. Samples were collected from the archives and each was given an identification number, connecting the sample to the clinical data and enabling anonymous analysis. In total, four 1.0‐mm cores were taken from each tumor sample and embedded in a new paraffin block using an automatic tissue microarray instrument (TMA Grand Master; 3D Histech Ltd). Samples were subsequently cut and processed in 4‐µm sections for immunohistochemistry.

The procedure was continued by fixation and drying of the slides (at 37°C for 12‐24 hours). Subsequently, sections were deparaffinized in xylene and rehydrated through lowering the concentrations of ethanol and distilled water. Slides were treated in a PT module (LabVision UK Ltd) in a Tris‐HCl buffer (pH 8.5) for 20 minutes at 98°C. Staining was carried out in an Autostainer 480 (LabVision) using the Dako REAL EnVision Detection System, Peroxidase/DAB+, Rabbit/Mouse (Dako). Next, the slides were incubated with a 0.3% Dako REAL Peroxidase‐Blocking Solution for 5 minutes to block the endogenous peroxidase activity. Then the samples were treated and incubated with a mouse monoclonal MMP14 antibody (diluted to 1:70 in a Dako REAL Antibody Diluent; Chemicon International, Inc) for 1 hour and with a peroxidase‐conjugated Dako REAL EnVision/HRP, Rabbit/Mouse reagent for 30 minutes. Visualization was completed using the Dako REAL DAB + Chromogen, and incubated for 10 minutes. Slides were counterstained using Meyer's hematoxylin, washed in tap water for 10 minutes, and mounted in Aquamount (BDH).

The same protocol was used for the PROX1 staining, except for the detection kit (ImmPRESS HRP Polymer Detection Kit, Peroxidase, anti‐goat IgG; Vector Laboratories) and the antibody used (anti‐human Prox1 antibody, diluted to 1:1800 = 18 µg/mL incubated overnight at room temperature [RT]; R&D Systems, Inc).^18^

### 2.3 Scoring of immunoreactivities

**Figure 1:**
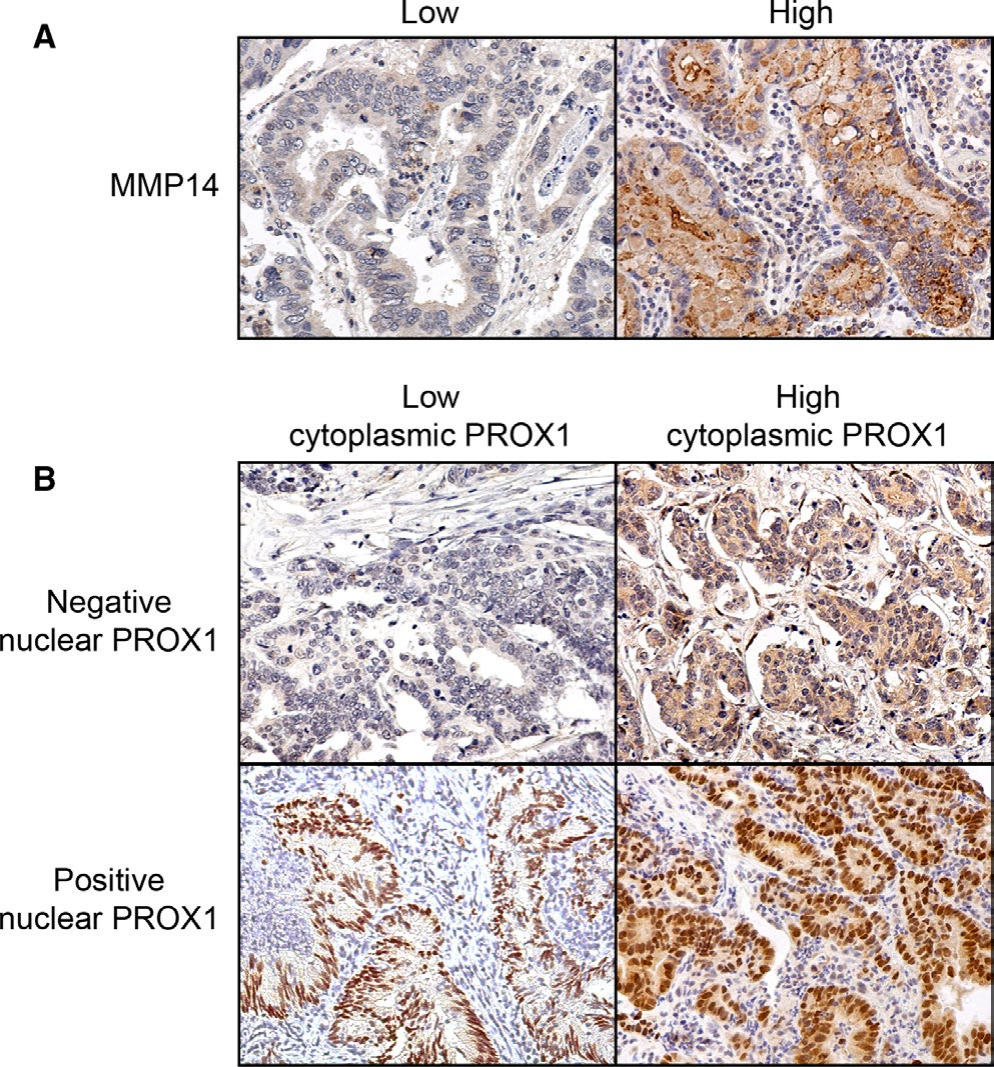
Representative images of the immunohistochemistry of gastric cancer tumors with (A) matrix metalloproteinase 14 (MMP14) staining and (B) prospero homeobox protein 1 (PROX1) staining. Original magnification at 20×

In total, 278 tissue cores in the MMP14‐stained samples included cancer cells and were suitable for the interpretation of MMP14 expression (Figure 1A). Matrix metalloproteinase 14 was distributed in the intracellular perinuclear cytoplasmic vesicles and diffusely on the cancer cells' plasma membrane. We quantified the MMP14 expression by scoring the cancer cells' cytoplasmic staining intensity, where a score of 3 indicated strong staining, 2 indicated moderate, 1 indicated weak, and 0 indicated absent staining. For the final analyses, the data were split in two, between a high expression (consisting of scores 2 and 3) and a low expression (consisting of scores 0 and 1) group. In addition, 275 tissue cores in the PROX1‐stained samples were suitable for scoring the amount of PROX1 positive nuclei (Figure 1B). PROX1 staining was observed in the cytoplasm and in the nucleus. PROX1‐stained samples were scored quantitatively for the number of cells harboring nuclear PROX1, where 4 represented over 75%, 3 represented 50%‐75%, 2 represented 25%‐50%, 1 represented less than 25%, and 0 represented no reactivity in the nucleus. For the final analyses, the data were grouped into two categories: negative (consisting of samples with no nuclear PROX1 staining) and positive (consisting of samples with any nuclear PROX1 staining) groups. The highest score for the four tissue microarray cores per patient was chosen to represent the patient in the statistical analyses. All samples were scored by two researchers (AK and JH), who were blinded to the clinical data. Cases with any variance were re‐assessed and the final score was reached through consensus.

### 2.4 Cell culture

We used 11 human gastric cancer cell lines: AGS (gastric adenocarcinoma), MKN‐28, MKN‐7, MKN‐74 (intestinal‐type gastric adenocarcinoma), TMK‐1, OCUM‐2MD3, OCUM‐12, OCUM‐14, NUGC3, MKN‐45, and KATOIII (diffuse‐type gastric adenocarcinoma).^24^, ^25^, ^26^, ^27^, ^28^, ^29^ The KATOIII and MKN‐7 cell lines were purchased from the Japanese Collection of Research Bioresources' cell bank (JCRB Cell Bank, National Institutes of Biomedical Innovation, Health and Nutrition), the MKN‐28, TMK‐1, and AGS cell lines were kindly provided by Professor Ari Ristimäki (University of Helsinki), and Professor Masakazu Yashiro (Osaka City University Graduate School of Medicine) provided MKN‐74, MKN‐45, NUGC3, OCUM‐14, OCUM‐12, and OCUM‐2MD3 cell lines. The cells were grown in an RPMI‐1640 media supplemented with 10% fetal calf serum (Gibco), 1% L‐Glutamine, and 1% Pen Strep (Thermo Fisher Scientific). Cells were grown in standard conditions (37°C, 5% CO_2_).

### 2.5 Small interfering RNA transfection

AGS cells were plated at a density of 2 × 10^5^ cells/mL. On the following day, the cells were transfected with either a control (12935200; Invitrogen) or two different PROX1‐targeting small interfering RNAs (siRNAs) (Stealth RNAi™ targeting HSS 108596; HSS 108597; Invitrogen) using Lipofectamine RNAiMAX (Thermo Fisher Scientific) according to the manufacturer's instructions. Two days after transfection, the cells were harvested and lysed for immunoblot analysis.

### 2.6 Protein detection by immunofluorescence assay

AGS, TMK‐1, MKN28, and MKN7 cells were plated on cover slips in a 24‐well plate, with 10^5^ cells placed in each well in 500 µL of RPMI‐1640 media. Cells were fixed by cross‐linking with 4% paraformaldehyde (diluted in phosphate buffered saline [PBS]), and incubated for 20 minutes at RT. Cells were permeabilized with 0.3% triton‐X (diluted in PBS) and simultaneously the nuclei were stained with Hoechst 1 µg/mL, incubated for 10 minutes at RT, and washed with PBS. Blocking was completed by incubating the cover slips for 45 minutes at RT in 0.5% bovine serum albumin (BSA) in PBS. The primary antibodies used were rabbit anti‐PROX1 (ab199359, diluted to 1:300 in 0.5% BSA in PBS; Abcam) and rabbit anti‐MMP14 (ab51074, diluted to 1:100 in 0.5% BSA in PBS; Abcam). Cells were incubated for 1 hour in wet chambers at RT with the primary antibody, and subsequently washed with PBS. The secondary antibody used was goat anti‐rabbit Alexa Fluor 594‐conjugated (diluted to 1:500 in 0.5% BSA in PBS). Incubation for 1 hour at RT took place in wet chambers. Finally, cells were washed with PBS and distilled water, then mounted on slides with Mowiol 4‐88 and incubated overnight at RT. Cover slips were imaged using the Sigma Panoramic FLASH II, 3DHIstech‐Aldrich, 81381.

### 2.7 Protein lysis and immunoblot

Cell pellets were produced by centrifuging 1 million cells of each cell line. The cells were treated with 200 µL of a radioimmunoprecipitation assay buffer (150 mmol/L NaCl, 1% NP‐40, 0.5% Na‐deoxycholate, 0.1% sodium dodecyl sulfate, and 50 mmol/L Tris‐HCl pH 8.0), supplemented with the phosphatase (Pierce™ 88667) and protease (Pierce™ 88666) inhibitors. Lysis was performed alternating 3 seconds vortexing with 10 minutes on ice for a total of 30 minutes. Cell lysate was cleared by centrifugation (16 000 *g*, for 20 minutes at 4°C). Cell lysate was boiled for 10 minutes with SDS sample buffer and reducing agent and loaded onto Criterion TGX precast gels (Bio‐Rad). Gels were run for 45 minutes at 55 mA and transferred on to nitrocellulose membranes with the trans‐blot Turbo Transfer system (Bio‐Rad). Blocking and antibody incubations were performed in a 5% nonfat milk, Tris‐buffered saline, and 0.01% tween‐100 solution for 1 hour at RT. Membranes were incubated in a rabbit monoclonal anti‐PROX1 antibody (ab199359, diluted to 1:1000; Abcam) or a rabbit monoclonal anti‐MMP14 antibody (ab51074, diluted to 1:1000; Abcam), and a mouse monoclonal anti‐beta‐actin antibody (sc‐47778, diluted to 1:2500; Santa Cruz Biotechnology) or a mouse anti‐TBG1 antibody (T6557; Sigma‐Aldrich) at 4°C overnight with gentle rocking. After washing, membranes were incubated in the appropriate secondary antibody (anti‐rabbit HRP‐linked antibody (7074; CST) and the anti‐mouse HRP‐linked antibody (7076; CST) diluted to 1:2500. Chemiluminescent detection was carried out through a 1‐minute incubation with the WesternBright Sirius HRP substrate components mixed at 1:1 (K‐12043‐D20; Advansta Corporation) at RT with gentle shuffling. The membrane was imaged in a Chemi Doc XRS+ (Bio‐Rad).

### 2.8 Quantification of the immunoblot band intensities

The band intensities were quantified using the Fiji software program (https://imagej.net/Fiji) and normalized to the corresponding loading control. The numbers below each blot indicate the relative band intensity compared to the control.

### 2.9 Statistical analyses

We calculated the *P* values for associations and correlations between PROX1, MMP14, and clinicopathologic variables using the Pearson's chi‐squared test and the Spearman's rank correlation test. Survival curves were created using the Kaplan‐Meier method and the *P* values were determined based on the log‐rank test. The Cox proportional hazard model was applied to the univariate and multivariate survival analyses. Covariates entered into the multivariate survival analyses consisted of age, cancer staging, the Laurén classification, and MMP14 expression. The cancer staging was processed as a categorical covariate. We found no significant interaction terms. Disease‐specific survival was calculated from the date of surgery until death due to gastric cancer. For all statistical analyses, we considered *P* < .05 as statistically significant. All statistical analyses were performed using IBM's SPSS Statistics, version 24.0 for Mac (IBM Corporation).

## 3 RESULTS

### 3.1 Associations of MMP14 and PROX1 expression with clinicopathologic variables

Among 278 cases suitable for analysis, 26 (9.4%) had a strong, 54 (19.4%) a moderate, and 83 (29.9%) had a weak MMP14 expression. No expression was found in 115 (41.4%) samples. A high MMP14 expression was associated with an older age (*P* = .041; Table 1).

**Table 1:** Association of MMP14 and PROX1 expressions with clinicopathologic variables in 278 gastric cancer patients

	MMP14^a^			Nuclear PROX1^a^		
	Low (%)	High (%)	*P* value^b^	Negative (%)	Positive (%)	*P* value^b^
Age, y						
<67	106 (76.8)	32 (23.2)	.041	81 (59.1)	56 (40.9)	.298
≥67	92 (65.7)	48 (34.3)		73 (52.9)	65 (47.1)	
Gender						
Male	103 (75.7)	33 (24.3)	.104	77 (57.0)	58 (43.0)	.734
Female	95 (66.9)	47 (33.1)		77 (55.0)	63 (45.0)	
Stage						
I	39 (76.5)	12 (23.5)	.821	22 (44.0)	28 (56.0)	.254
II	46 (70.8)	19 (29.2)		36 (55.4)	29 (44.6)	
III	72 (69.9)	31 (30.1)		63 (61.2)	40 (38.8)	
IV	40 (69.0)	18 (31.0)		32 (57.1)	24 (42.9)	
Tumor classification (pT)						
pT1	33 (80.5)	8 (19.5)	.251	17 (42.5)	23 (57.5)	.312
pT2	31 (77.5)	9 (22.5)		24 (60.0)	16 (40.0)	
pT3	56 (65.1)	30 (34.9)		50 (58.8)	35 (41.2)	
pT4	78 (70.3)	33 (29.7)		63 (57.3)	47 (42.7)	
Lymph node metastasis (pN)						
pN0	63 (70.8)	26 (29.2)	.969	45 (51.1)	43 (48.9)	.304
pN1‐3	127 (70.6)	53 (29.4)		104 (57.8)	76 (42.2)	
Distant metastasis (pM)						
M0	158 (71.8)	62 (28.2)	.669	122 (55.7)	97 (44.3)	.847
M1	40 (69.0)	18 (31.0)		32 (57.1)	24 (42.9)	
Laurén classification						
Intestinal	81 (72.3)	31 (27.7)	.740	71 (63.4)	41 (36.6)	.041
Diffuse	117 (70.5)	49 (29.5)		83 (50.9)	80 (49.1)	

Abbreviations: MMP14, matrix metalloproteinase 14; PROX1, prospero homeobox protein 1.

a By immunohistochemistry.

b Pearson chi‐squared test.

Over 75% nuclear PROX1 expression was observed in 10 samples (3.6%), 50% to 75% in 12 samples (4.4%), 25% to 50% in 28 samples (10.2%), and less than 25% in 71 samples (25.8%). Among the 275 cases, in which PROX1 staining was interpretable in, nuclear PROX1 expression was absent in 154 (56.0%) samples. Positive nuclear PROX1 expression was associated with diffuse‐type tumors (*P* = .041; Table 1) and with a high cytoplasmic PROX1 expression (*P* < .001; Table 2). We also found a weak positive correlation between the nuclear and cytoplasmic PROX1 expressions (*r*_s_ = .310; *P* < .001). The association between the nuclear and high cytoplasmic PROX1 expressions was examined separately in intestinal and diffuse‐type tumors, for which we found strong associations in both (Table S1).

**Table 2:** Association between MMP14 and PROX1 expressions in 278 gastric cancer patients

	MMP14^a^			Nuclear PROX1^a^		
	Low (%)	High (%)	*P* value^b^	Negative (%)	Positive (%)	*P* value^b^
Serum MMP14						
Low	127 (70.9)	52 (29.1)	.804	92 (52.3)	84 (47.7)	.273
High	27 (73.0)	10 (27.0)		23 (62.2)	14 (37.8)	
Cytoplasmic PROX1^a^						
Low	153 (71.5)	61 (28.5)	.992	137 (64.6)	75 (35.4)	<.001
High	40 (71.4)	16 (28.6)		15 (26.8)	41 (73.2)	
Nuclear PROX1^a^						
Negative	109 (71.2)	44 (28.8)	.904			
Positive	87 (71.9)	34 (28.1)				

Abbreviations: MMP14, matrix metalloproteinase 14; PROX1, prospero homeobox protein 1.

a By immunohistochemistry.

b Pearson chi‐squared test.

### 3.2 Survival analyses

**Figure 2:**
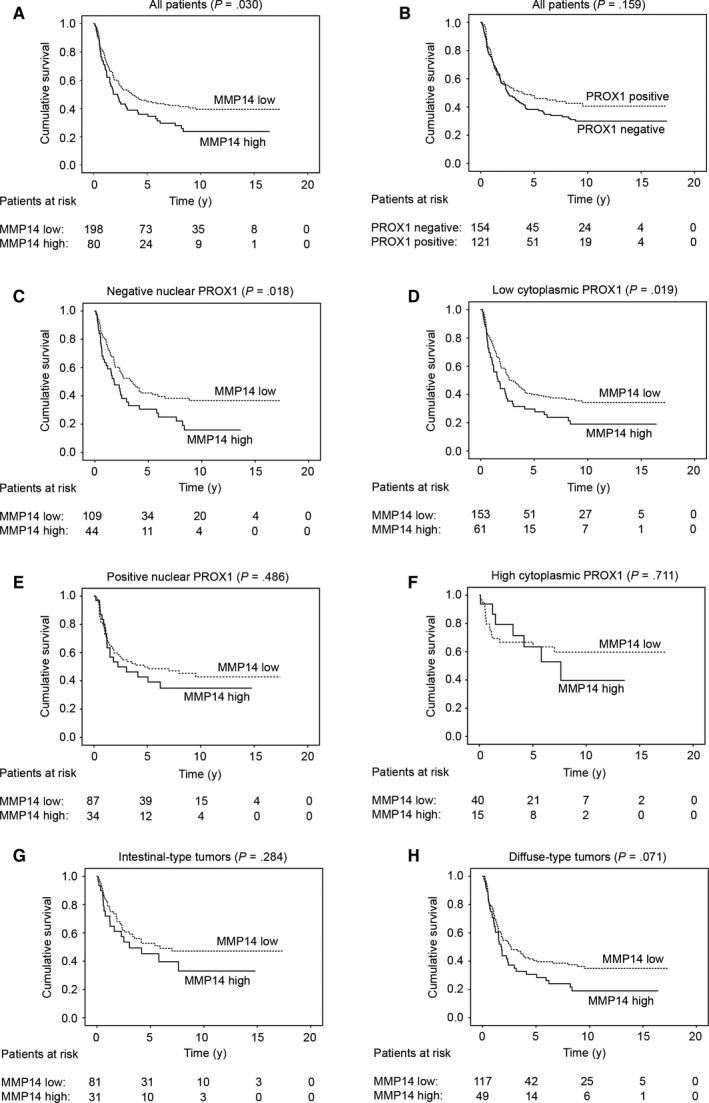
Disease‐specific survival of gastric cancer patients according to the Kaplan‐Meier method. A, Low versus high matrix metalloproteinase 14 (MMP14) expression. B, Negative vs positive nuclear prospero homeobox protein 1 (PROX1) expression. Low vs high MMP14 expression among patients with (C) a negative nuclear PROX1 expression, (D) a low cytoplasmic PROX1 expression, (E) a positive nuclear PROX1 expression, (F) a high cytoplasmic PROX1 expression, (G) intestinal‐type, and (H) diffuse‐type tumors. *P* value calculated using the log‐rank test

Five‐year disease‐specific survival for gastric cancer patients with a low MMP14 expression was 45.3% (95% CI 38.0‐52.6), whereas for patients with a high MMP14 it was 35.9% (95% CI 24.9‐46.9; *P* = .030; Figure 2A). Gastric cancer patients with a high MMP14 expression had a hazard ratio (HR) of 1.43 (95% CI 1.03‐1.98; *P* = .031; Table 3) for the disease‐specific survival. Nuclear PROX1 expression did not emerge as a significant prognostic factor (Figure 2B; Table 3).

**Table 3:** Uni‐ and multivariate survival analyses for gastric cancer patients according to the Cox proportional hazards model

	Univariate survival analysis			Multivariate survival analysis		
	HR	95% CI	*P* value	HR	95% CI	*P* value
Age, y						
<67	1.00			1.00		
≥67	1.33	1.00‐1.79	.054	2.50	1.79‐3.48	<.001
Stage						
I	1.00			1.00		
II	5.44	2.25‐13.1	<.001	4.51	1.72‐11.9	.002
III	15.7	6.85‐36.1	<.001	16.6	6.67‐41.1	<.001
IV	46.2	19.6‐109	<.001	62.8	24.4‐161	<.001
Laurén classification						
Intestinal	1.00			1.00		
Diffuse	1.45	1.06‐1.98	.020	1.62	1.16‐2.27	.005
MMP14^a^						
Low	1.00			1.00		
High	1.43	1.03‐1.98	.031	1.31	0.94‐1.82	.110
Nuclear PROX1^a^						
Negative	1.00					
Positive	0.80	0.58‐1.09	.160			

Abbreviations: CI, confidence interval; HR, hazard ratio; MMP14, matrix metalloproteinase 14; PROX1, prospero homeobox protein 1.

a By immunohistochemistry.

In the subgroup analyses, a high MMP14 expression emerged as a marker of a worse prognosis among patients with negative nuclear PROX1 expression (Figure 2C) and among those with a low cytoplasmic PROX1 expression (Figure 2D), but not among patients with a positive nuclear PROX1 expression (Figure 2E), a high cytoplasmic PROX1 expression (Figure 2F), intestinal‐ (Figure 2G) or diffuse‐type tumors (Figure 2H; Table 4). In addition, a high MMP14 level indicated a worse prognosis among patients with pT3 tumors, among those with lymph node metastases, and among those without distant metastases (Table 4).

**Table 4:** Survival analyses by subgroups for gastric cancer patients according to the Cox proportional hazards model

	High MMP14^a^			Positive nuclear PROX1^a^		
	HR	95% CI	*P* value	HR	95% CI	*P* value
Age, y						
<67	1.56	0.95‐2.56	.081	0.63	0.39‐1.02	.060
≥67	1.23	0.80‐1.90	.349	0.94	0.61‐1.44	.775
Gender						
Male	1.55	0.95‐2.54	.082	0.61	0.38‐0.98	.043
Female	1.33	0.86‐2.06	.196	1.02	0.67‐1.56	.930
Stage						
I	0.93	0.10‐8.31	.947	1.36	0.23‐8.15	.738
II	1.73	0.77‐3.90	.185	0.96	0.42‐2.09	.870
III	1.39	0.87‐2.23	.166	0.84	0.53‐1.32	.448
IV	1.25	0.70‐2.25	.447	0.98	0.56‐1.72	.938
Tumor classification (pT)						
pT1	1.65	0.17‐15.9	.666	2.43	0.25‐23.4	.442
pT2	0.55	0.12‐2.53	.443	0.71	0.21‐2.36	.576
pT3	1.74	1.04‐2.92	.036	0.60	0.35‐1.03	.066
pT4	1.03	0.65‐1.64	.892	1.07	0.70‐1.64	.763
Lymph node metastasis (pN)						
pN0	1.63	0.74‐3.55	.223	0.47	0.21‐1.06	.068
pN1‐3	1.52	1.06‐2.20	.025	1.02	0.71‐1.45	.931
Distant metastasis (pM)						
pM0	1.51	1.02‐2.23	.042	0.74	0.50‐1.08	.116
pM1	1.25	0.70‐2.25	.447	0.98	0.56‐1.72	.938
Laurén classification						
Intestinal	1.37	0.77‐2.43	.286	0.72	0.40‐1.29	.266
Diffuse	1.44	0.97‐2.14	.072	0.76	0.52‐1.12	.165
MMP14^a^						
Low				0.89	0.60‐1.30	.538
High				0.67	0.39‐1.18	.166
Serum MMP14						
Low	1.42	0.94‐2.15	.099	0.77	0.51‐1.15	.200
High	1.42	0.63‐3.23	.401	0.83	0.39‐1.79	.637
Cytoplasmic PROX1^a^						
Low	1.53	1.07‐2.18	.020	1.03	0.72‐1.46	.889
High	1.19	0.48‐2.91	.711	0.60	0.25‐1.43	.247
Nuclear PROX1^a^						
Negative	1.65	1.09‐2.51	.019			
Positive	1.21	0.71‐2.06	.487			

Abbreviations: CI, confidence interval; HR, hazard ratio; MMP14, matrix metalloproteinase 14; PROX1, prospero homeobox protein 1

a By immunohistochemistry.

Furthermore, a positive nuclear PROX1 expression served as a marker of a better prognosis in men (Table 4). We found a significantly better prognosis among patients with high cytoplasmic and positive nuclear PROX1 expressions (HR 0.45; 95% CI 0.26‐0.78; *P* = .004). Among patients with high cytoplasmic and positive nuclear PROX1 expressions, 5‐year survival was 68.6% (95% CI 54.1‐83.5), whereas among patients with low cytoplasmic and no nuclear PROX1 expressions 5‐year survival fell to 37.9% (95% CI 31.2‐44.6; *P* = .003).

### 3.3 Immunofluorescence, immunoblot, and siRNA transfection analyses

**Figure 3:**
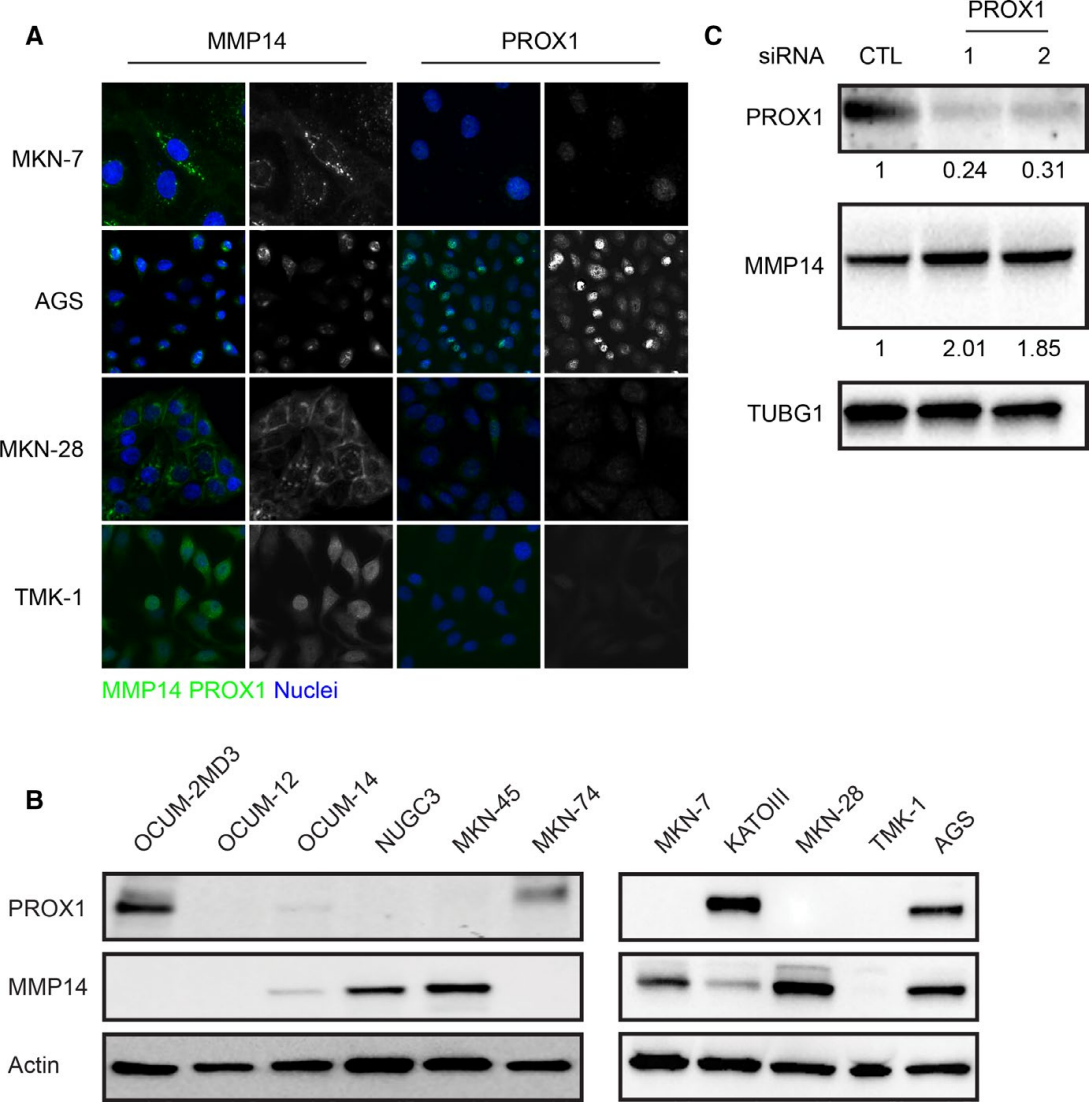
Immunofluorescence, immunoblot, and small interfering RNA (siRNA) transfection analyses for gastric cancer cell lines. A, Immunofluorescence analysis of gastric cancer cell lines. Green indicates matrix metalloproteinase 14 (MMP14) and prospero homeobox protein 1 (PROX1), blue indicates the nuclei. B, Immunoblot analysis for gastric cancer cell lines. C, siRNA transfection of the AGS cells. The AGS cells were transfected with the indicated siRNA, and analyzed 48 h later by immunoblot for the indicated antibody. TUBG1 was used as the loading control. The values indicate the relative PROX1 and MMP14 band intensities normalized to the corresponding loading control

PROX1 was recently shown to suppress MMP14 expression in breast cancer, as well as hepatocellular and colorectal carcinoma; here we showed that survival is worse specifically among those gastric cancer patients with a high MMP14 and a low PROX1 expression.^21^ To further explore the connection between MMP14 and PROX1 in gastric cancer, we proceeded by studying the expression levels in cell lines. Cell lines, intestinal MKN‐7, intestinal MKN‐28, diffuse TMK‐1, and AGS (unknown histological type) were studied using immunofluorescence (Figure 3A). In MKN‐7 and AGS, a weak MMP14 expression was observed, while in MKN‐28 and TMK‐1 expression was stronger. In MKN‐7, MKN‐28, and TMK‐1, PROX1 expression was very weak, but stronger in AGS. In these cell lines, MMP14 was expressed perinuclearly and on the cell surface, whereas PROX1 expression was nuclear. Variable levels of MMP14 expression in MKN‐7, MKN‐28, and TMK‐1 accompanied by a nearly absent PROX1 expression may indicate a functional connection between MMP14 and PROX1 in gastric cancer as well, although in AGS both MMP14 and PROX1 were expressed.

In addition, we studied gastric cancer cells using the immunoblot analysis (Figure 3B). The predicted molecular weight for PROX1 is 83 kDa, whereas for MMP14 it is 66 kDa. Among the intestinal‐type cell lines, MMP14 was expressed in MKN‐28 and MKN‐7, in which PROX1 was not expressed. Furthermore, in the MKN‐74 cells, PROX1 was expressed while MMP14 expression was absent, supporting the inverse correlation in their expression as recently demonstrated by Gramolelli et al.^21^ Among the diffuse cell lines, OCUM‐2MD3 cells expressed PROX1, but not MMP14, whereas the NUGC3 and MKN‐45 cells expressed MMP14, but lacked the PROX1 expression. In the KATOIII cells, PROX1 was strongly expressed and MMP14 expression was lower than in MKN‐7, MKN‐28, NUGC3, and MKN‐45, further supporting the negative regulation of MMP14 by PROX1. In the gastric adenocarcinoma cell line, AGS, MMP14, and PROX1 were both expressed, but did not show clear inverse expression levels. In the OCUM‐14 cells, MMP14 and PROX1 were both very weakly expressed. The OCUM‐12 or TMK‐1 cells expressed neither PROX1 nor MMP14. In total, seven out of eleven gastric cancer cell lines—MKN‐28, KATOIII, MKN‐7, MKN‐74, MKN‐45, NUGC3, and OCUM‐2MD3—showed a clear inverse correlation with the MMP14 and PROX1 expression levels.

To further explore the functional connection between MMP14 and PROX1 in gastric cancer, we silenced PROX1 in the AGS cells using two different siRNAs. Compared to the control siRNA‐treated cells, using both PROX1‐targeting siRNAs we achieved an efficient reduction in the PROX1 level (76% and 69%, respectively), while the MMP14 protein levels increased by 2.01‐ and 1.85‐fold, respectively, as shown by immunoblotting (Figure 3C).

## 4 DISCUSSION

In this study, we confirm that survival is worse among gastric cancer patients with a high MMP14 tissue expression and, for the first time, show that survival is worse particularly when PROX1 is low. We also found that nuclear PROX1 expression in gastric cancer tissue samples associates with diffuse‐type tumors. In addition, by including data from a previous study of cytoplasmic PROX1 expression in gastric cancer tissue samples from the same patient cohort, we discovered that 5‐year disease‐specific survival among patients with positive nuclear and high cytoplasmic expressions reached nearly 70%.^18^

Our results agree with previous studies of MMP14 in gastric cancer and provide further validation that survival is worse among gastric cancer patients with a high tissue MMP14 expression.^5^, ^9^, ^10^ Here, survival was worse particularly among patients with lymph node‐metastases, no distant metastases, pT3 tumors, and importantly, among patients with either no nuclear or low cytoplasmic PROX1 immunoreactivity. This supports the prominent role played by MMP14 in cancer metastasis indicating that a low or absent PROX1 expression may be required for MMP14 to be highly expressed. This finding agrees with the previously described suppressive role of PROX1 on MMP14 transcription.^21^

PROX1 was recently shown to regulate the transcription of MMP14 in multiple cellular contexts; however, gastric cancer cases were not included in that study.^21^ Here, we explored the connection between PROX1 and MMP14 in gastric cancer, revealing inversely correlated expression levels in seven of the eleven cell lines studied, suggesting that the PROX1‐MMP14 regulatory axis may also be functional in gastric cancer. Additionally, in the immunofluorescence analysis, the MMP14 expression in MKN‐7 and MKN‐28 accompanied by a nearly absent PROX1 expression may indicate a functional connection between MMP14 and PROX1 in gastric cancer as well. In the AGS cells, however, MMP14 and PROX1 co‐expressed, indicating that other PROX1‐independent mechanisms may also be involved. We then silenced PROX1 in the AGS cells to further explore the connection between MMP14 and PROX1 in gastric cancer, finding that siRNA transfection using two PROX1‐targeting siRNAs resulted in increased MMP14 protein levels in both cases. This further indicates that PROX1 participates in governing the MMP14 expression in gastric cancer as well.

Few studies of PROX1 in gastric cancer have been published, reporting inconsistent results, showing that it is highly expressed in cancer compared to healthy tissue and associates with a worse although paradoxically with a better patient prognosis.^17^, ^18^, ^19^, ^20^ On the one hand, Ueta et al^20^ concluded that nuclear PROX1 expression associates with a worse prognosis. On the other hand, Laitinen et al^18^ found that cytoplasmic PROX1 expression associated with a better prognosis. Here we focused on the nuclear PROX1 expression, since PROX1 acts as a transcription factor. We found no significant association between nuclear PROX1 expression and survival in the entire patient cohort; however, when combining the results of nuclear PROX1 expression to previous results on cytoplasmic PROX1 expression, we found that taking nuclear PROX1 expression into account strengthens the association of PROX1 tissue expression with a better prognosis. This stands in contrast to the oncogenic role of PROX1 previously found in gastric cancer and with the presumed connection between the nuclear PROX1 expression and a worse prognosis. To clarify whether cytoplasmic PROX1 merely originates from the nucleus or if it has some distinct extra‐nuclear function in gastric cancer, further research is necessary.

The strengths of this study include the large patient cohort with reliable follow‐up information, validation of previous results, and the exploration of the roles of MMP14 and PROX1 in gastric cancer in both laboratory and clinical settings. More extensive laboratory experiments and analysis on other large, well‐defined patient cohorts are needed to further disentangle the functions and cross talk between MMP14 and PROX1 in gastric cancer. Since this study was carried out retrospectively and, thus, accessing patient details may introduce inaccuracies, certain well‐known risk factors in gastric cancer, such as venous and perineural invasion and lymphatic emboli, were not included in our analyses.

In conclusion, this study confirms that a high MMP14 expression predicts worse survival in gastric cancer and, for the first time, shows that survival is worse if PROX1 specifically is low.

## Data Availability

All data and materials are available from the corresponding author upon request.
