# Supplementary material for: High tissue MMP14 expression predicts worse survival in gastric cancer, particularly with a low PROX1
Source: Cancer Med. 2019 Sep 27;8(16):6995–7005. doi: 10.1002/cam4.2576 (PMC6853825; doi:10.1002/cam4.2576)
Supplement: Supplementary file 1 [file CAM4-8-6995-s001.docx]

**Supplementary Table 1.** Association of nuclear PROX1 expression with cytoplasmic PROX1 and MMP14 expressions in 112 patients with intestinal and 163 with diffuse type of gastric cancer.

|  |  |  |  |  |  |  |  |
| --- | --- | --- | --- | --- | --- | --- | --- |
|  | **Intestinal** | | |  | **Diffuse** | | |
|  | **Nuclear PROX1^†^** | | |  | **Nuclear PROX1^†^** | | |
|  | **Negative (%)** | **Positive (%)** | ***P* value*** |  | **Negative (%)** | **Positive (%)** | ***P* value*** |
|  |  |  |  |  |  |  |  |
|  |  |  |  |  |  |  |  |
| MMP14^†^ |  |  |  |  |  |  |  |
| Low | 52 (64.2) | 29 (35.8) | 0.775 |  | 57 (49.6) | 58 (50.4) | 0.675 |
| High | 19 (61.3) | 12 (38.7) |  |  | 25 (53.2) | 22 (46.8) |  |
|  |  |  |  |  |  |  |  |
| Cytoplasmic PROX1^†^ |  |  |  |  |  |  |  |
| Low | 58 (75.3) | 19 (24.7) | <0.001 |  | 79 (58.5) | 56 (41.5) | <0.001 |
| High | 13 (38.2) | 21 (61.8) |  |  | 2 (9.1) | 20 (90.9) |  |
|  |  |  |  |  |  |  |  |

Abbreviations: PROX1 = Prospero homeobox protein 1, MMP14 = Matrix metalloproteinase 14

† By immunohistochemistry, * Pearson Chi–square test
